# C-953-02. Denial Rates of Burn Scar Laser Treatment over Time, a Retrospective Review

**DOI:** 10.1093/jbcr/irag033.126

**Published:** 2026-04-06

**Authors:** Matthew D Supple, Jonathan S Friedstat, Daniela Requena, Crystal New, Sharon M Dell'Anno, John T Schulz, Jeremy Goverman

**Affiliations:** Massachusetts General Hospital, Boston, MA; Massachusetts General Hospital, Boston, MA; Massachusetts General Hospital, Boston, MA; Massachusetts General Hospital, Boston, MA; Massachusetts General Hospital, Boston, MA; Massachusetts General Hospital, Boston, MA; Massachusetts General Hospital, Boston, MA

## Abstract

**Introduction:**

Laser therapy is an effective treatment at improving outcomes for burn survivors from symptomatic and hypertrophic burn scars. Despite lasers being increasingly adopted by burn centers, we noticed more patients were denied laser treatment coverage by their insurance carriers. With reasonable medical documentation, photographs, literature supporting the rationale for treatment and prior authorizations, we were puzzled why this was happening. In 2024, we began to utilize t codes for laser submissions and our hypothesis was that decreases in laser payment was related to this coding change. Therefore, we performed a retrospective review of the claims data from 2023 and 2024 to evaluate rates of payment for laser therapy over time with use of the t-codes.

**Methods:**

With IRB approval, insurance claim data was pulled from fiscal year 2023 and 2024 for laser treatment of burn patients. In 2023, the laser codes were 17 106-8. In 2024, they were 17 106-8, 0479 t, and 0480 t. As a negative control, we also compared reconstructive surgical codes for adjacent tissue rearrangements and full thickness skin grafts. Demographic information was collected for both groups (age, sex, TBSA, etiology, and ethnicity). Rates of insurance approval were noted for both groups over time. Laser and reconstructive surgical procedures were compared over time and to one another and for rates of approval. T-tests with unequal variance were performed.

**Results:**

Comparing laser and surgical reconstructive treatments, the demographics of the two groups were very similar, as patients often were in both, but a few differences were noted. Males had surgery 8% more than females (*p*=.041), females had laser 15% more than males (*p*=.035), and patients with a TBSA under 10% had laser 14% more than surgery (*p*=.006).

From 2023 to 2024, laser payment rates decreased from 94.3% to 78.2% (*p* = < 0.001). In 2024 when t-codes were first used, 13 were paid, 20 were denied and there was no difference between these groups (*p*=.21). Reconstructive procedures were paid at 94.5% in 2023 and 94.4% in 2024 (*p*=.98). Laser vs. reconstructive surgery showed no difference in payment rates in 2023 and 2024, both at 94% (*p*=.94), but in 2024 there was a significant difference in payment of 78.9% vs. 94.4% (*p*<.001) respectively.

**Conclusions:**

Based on our analysis from 2023 to 2024, the 16.1% decrease in payment for laser was not explained by demographic differences or the use of t codes, and a similar decrease was not seen in reconstructive surgical procedures. Further research is needed to better explain the decline in payment rates by insurance carriers for laser.

**Applicability of Research to Practice:**

We plan to analyze fiscal year 2025 for laser and surgical reconstructive surgeries to trend the data over a three-year timeframe, with a full year of t-code utilization. By evaluating laser denial rates, we hope to better understand these insurance challenges so we can improve access to laser therapy for burn survivors.

**Funding for the study:**

N/A.

